# *Esrp1* is a marker of mouse fetal germ cells and differentially expressed during spermatogenesis

**DOI:** 10.1371/journal.pone.0190925

**Published:** 2018-01-11

**Authors:** Shaghayegh Saeidi, Farnaz Shapouri, Robb U. de Iongh, Franca Casagranda, Jessie M. Sutherland, Patrick S. Western, Eileen A. McLaughlin, Mary Familari, Gary R. Hime

**Affiliations:** 1 Department of Anatomy and Neuroscience, University of Melbourne, Parkville, Australia; 2 School of Biomedical Sciences and Pharmacy, University of Newcastle, Callaghan, Australia; 3 Centre for Genetic Diseases, Hudson Institute of Medical Research and Department of Molecular and Translational Science, Monash University, Clayton, Australia; 4 School of Environmental and Life Sciences, University of Newcastle, Callaghan, Australia; 5 School of Biological Sciences, University of Auckland, Auckland, New Zealand; 6 School of Biosciences, University of Melbourne, Parkville, Australia; National Cancer Institute, UNITED STATES

## Abstract

ESRP1 regulates alternative splicing, producing multiple transcripts from its target genes in epithelial tissues. It is upregulated during mesenchymal to epithelial transition associated with reprogramming of fibroblasts to iPS cells and has been linked to pluripotency. Mouse fetal germ cells are the founders of the adult gonadal lineages and we found that *Esrp1* mRNA was expressed in both male and female germ cells but not in gonadal somatic cells at various stages of gonadal development (E12.5-E15.5). In the postnatal testis, *Esrp1* mRNA was highly expressed in isolated cell preparations enriched for spermatogonia but expressed at lower levels in those enriched for pachytene spermatocytes and round spermatids. Co-labelling experiments with PLZF and c-KIT showed that ESRP1 was localized to nuclei of both Type A and B spermatogonia in a speckled pattern, but was not detected in SOX9^+^ somatic Sertoli cells. No co-localization with the nuclear speckle marker, SC35, which has been associated with post-transcriptional splicing, was observed, suggesting that ESRP1 may be associated with co-transcriptional splicing or have other functions. RNA interference mediated knockdown of *Esrp1* expression in the seminoma-derived Tcam-2 cell line demonstrated that ESRP1 regulates alternative splicing of mRNAs in a non-epithelial cell germ cell tumour cell line.

## Introduction

Germ cells exhibit unique profiles of gene expression that distinguish them from somatic cells (reviewed in [1]) and utilise specific transcriptional regulators, which produce transcripts that differ from those observed in other tissues [2]. Transcript diversity also derives from an extensive array of post-transcriptional regulation that is present in differentiating germ cells including extensive alternative splicing of pre-mRNA molecules that amplifies the number of proteins produced from a finite number of genes [3–8]. Genome-wide analyses of alternative splicing of transcripts in the gonads of *Drosophila* and mice, have demonstrated the existence of many germ-cell specific protein isoforms [8, 9] and a high frequency of alternate splicing events in the testis [10, 11]. The *Drosophila* study also identified RNA splicing factors that are highly enriched in pre-meiotic cells [9]. While the core elements of the RNA splicing mechanism are ubiquitously expressed and regulate mRNA splicing in all cells, splicing profiles differ between cells [12], suggesting that tissue specific regulators generate cell specific splicing events. In pursuit of this hypothesis, Warzecha et al. [13] conducted a genome wide screen to identify new factors that could uniquely promote splicing in epithelial cells. Among various factors, two protein paralogues were found to cause epithelial specific splicing patterns. Previously, these proteins were known as RNA binding motif proteins 35A and 35B (RBM35A and RBM35B). Expression of both genes is highly cell type specific, but up-regulation of both genes was generally observed in epithelial cell types. These proteins were thus renamed epithelial splicing regulatory proteins 1 and 2 (ESRP1 and ESRP2) [13].

Up-regulation of ESRP1 and ESRP2 expression coincides with the earliest changes in global gene expression associated with the mesenchymal to epithelial transition and induction of pluripotency during iPS cell generation [14, 15]. Moreover, a recent study of alternative splicing events, which occur during reprogramming of mouse embryonic fibroblasts to iPS cells, identified enrichment of ESRP1 binding sites upstream of alternatively spliced exons. Subsequent knockdown of ESRP1/2 followed by RNA-Seq analysis demonstrated that ESRP1/2 dependent splicing events occur during the induction of pluripotency [16].

Mouse spermatogonial stem cells, in addition to their capacity to repopulate germ cell-depleted seminiferous tubules [17], display pluripotent characteristics when isolated and cultured under the same conditions as embryonic stem cells [18–21] including expression of pluripotency markers (e.g. Oct4, Nanog, Rex-1), differentiation along mesodermal and neuroectodermal lineages, formation of teratomas when injected into SCID mice and generation of chimeras when injected into host blastocysts [18–21]. Similarly, pluripotent cells have been isolated from human testes [22, 23] but appear to be less competent or not as efficient as ES cells in forming chimeras and teratomas (reviewed in [24]). Comparison of rodent adult germline stem cells with ES cells by expression profiling demonstrated that they are almost identical, express the same level of pluripotency genes and respond similarly in differentiation assays [25].

Given the high level of alternate splicing during spermatogenesis and the association of ESRP1 with pluripotency, we were interested in examining the expression of ESRP1 during the development of male and female germ cells. Germ cells in the mouse are derived from a small number of cells present in the epiblast at E6.25 (embryonic day 6.25 after fertilization) that receive a BMP signal from extraembryonic ectoderm. After limited proliferation, these cells migrate, by both passive and actively directed transport and are found by E11.5 in the genital ridges, which are the gonadal precursors. By day E13.5 male fetal germ cells down regulate pre-meiotic genes, enter mitotic arrest and are termed gonocytes, whereas female embryonic germ cells differentiate into oogonia, which enter meiosis and arrest in prophase I. By birth, the majority of oogonia have either degenerated, or developed into primordial follicles, that remain in meiotic arrest unless selected for maturation during the adult female reproductive cycle. By contrast, gonocytes remain mitotically arrested until around post-natal day 3 in males, before re-entering the cell cycle and forming spermatogenic stem cells within the seminiferous tubules. The spermatogenic stem cells subsequently produce spermatogonia that will differentiate into spermatocytes. Each spermatocyte undergoes two meiotic divisions to produce four haploid round spermatids that undergo a morphological change to produce elongating spermatids, and eventually, mature spermatozoa (reviewed in [26]).

In this study we identify that *Esrp1* is transcribed in fetal germ cells and in perinatal gonocytes/spermatogonia and shows enriched expression in adult spermatogonia. In addition, we show that ESRP1 protein is most highly expressed in nuclei of pre-meiotic germ cells in adult testes and demonstrate that ESRP1 regulates alternative splice selection of mRNAs in a germ cell-derived cell line.

## Materials and methods

### Experimental animals

All animal procedures were approved by the Animal Ethics Committees of the University of Melbourne, the Murdoch Children’s Research Institute and the University of Newcastle. Mice (FVB, C57Bl6/J, CD1, and OG2) were maintained under standard housing conditions with *ad libitum* access to rodent chow and water and under a 12 hour dark, 12 hour light cycle. Wild-type mouse strains were obtained from ARC (Murdoch WA, Australia) and maintained as inbred colonies. OG2 mice (Jackson Lab Stock 004654), which express enhanced green fluorescent protein under the control of the *Pou5f1* promoter and distal enhancer [27] were maintained as homozygous transgenic strain on a C57Bl6/J background [28].

### Isolation of gonadal germ and somatic cells

Fetal gonadal germ and somatic cells were isolated as previously described [28, 29]. Briefly, fetal (E12.5 –E15.5) gonads were dissected and dissociated cells were fluorescent-activated cell sorting (FACS) to isolate GFP^+^ germ cells and GFP^-^ somatic cells. The identity of each isolated cell type was confirmed by immunofluorescence for specific germ cell (OCT4, MVH) and somatic cell (SOX9) markers [28].

Testes from 1 day (for isolation of gonocytes/spermatogonia), 8–10 day (spermatogonia) and 8 week (pachytene spermatocytes and round spermatids) old wild-type mice were dissociated using a combination of 0.5 mg/ml collagenase and 0.25% trypsin and separated on 2–4% continuous bovine serum albumin (BSA) gradient as described previously [30, 31]. RT-ddPCR was used to confirm the identities of the separated cell populations as described (*Ccnd1* and *Ngn3* were used to show enrichment of gonocytes/spermatogonia) [32], *Dnah8* showed enrichment of pachytene spermatocytes and Tob1 showed enrichment of round spermatids [29]. We obtained three independent biological replicates of enriched gonocytes, spermatogonia and pachytene spermatocytes but only two replicates of round spermatids.

### Cell culture and RNA interference

The TCam-2 cell line, which derives from a primary testicular seminoma [33] and exhibits characteristics of fetal germ cells [34] was cultured in RPMI 1640 medium (Thermo Fisher Scientific, Scoresby VIC, Australia) supplemented with 10% fetal calf serum (Life Technologies, Mulgrave VIC, Australia) and penicillin/streptomycin diluted 1:200 (Thermo Fisher Scientific) at 37°C in 5% CO_2_ in a humidified incubator.

To conduct *ESRP1* knockdown experiments, two *Silencer*^®^ siRNAs and a negative control were obtained from Ambion/ThermoFisher (Table 1). On the day prior to transfection, TCam-2 cells (~0.6 ×10^6^ cells/well) were seeded in RPMI medium in the absence of antibiotic in 6 well culture plates (3 per control and 3 per siRNA pair) (Nunclon^™^ Delta, ThermoFisher). The siRNA (25 ρmole) and 3% Lipofectamine RNAiMAX (Invitrogen/Life Technologies) diluted in Opti-MEM^®^ medium (Invitrogen/Life Technologies), as specified in the manufacturer’s instructions, were added to cells in each culture well. After 48 or 72 h of culture the cells were harvested and extracted for Western blotting.

**Table 1:** siRNA target sequences.

siRNA ID Silencer^®^Select	1. Sense Sequence (5’-3’) 2. Antisense Sequence (5’-3’)	Catalogue Number	Lot Number
**S29570**	1. GAGAGUGAAUUACAAGUUUTT 2. AAACUUGUAAUUCACUCUCTC	#4392420	# AS025SCB
**S29571**	1. CCUUCGAGGUCUUCCCUAUTT 2. AUAGGGAAGACCUCGAAGGCG	#4392420	# AS025SCC
**Negative Control**		#4390843	#AS023T9Q

### Cycloheximide chase assay

To investigate the stability of ESRP1 protein, TCam-2 cells were subjected to a cycloheximide chase assay. The transfection medium was replaced with RPMI medium containing 50 μg/ml cycloheximide (CHX) added into each well. At various time-points after addition of the cycloheximide (t = 24, 48, 72 hours), cells were harvested, centrifuged and lysed in RIPA buffer containing protease inhibitors for western blotting analysis.

### Western blot

Protein concentrations of cell lysates were determined using a bicinchoninic acid protein assay kit (BCA kit; ThermoFisher Scientific) and 10 μg of protein from each sample were electrophoresed on a 10% SDS PAGE gel and transferred onto an Immobilon-P PVDF membrane (Millipore, Australia). After blocking (5% milk powder and 0.05% Tween-20 in PBS) the membranes were incubated overnight at 4°C with anti-ESRP1 (HPA023719; Sigma; 1:250) and anti-β-actin (A5441, Sigma, 1:5000) antibodies, diluted in 5% BSA in PBS/Tween-20. After washes in PBS/Tween-20 the membranes were incubated for 1 h at room temperature with the appropriate HRP-conjugated secondary antibodies (Invitrogen) diluted in PBS/BSA/Tween-20. Signals were visualized using Clarity TM Western ECL substrates (BioRad) according to the manufacturer’s recommended protocol and imaged with a ChemiDocTM MP system (Bio-Rad).

### RNA extraction, cDNA synthesis, RT-PCR and droplet digital PCR (ddPCR)

RNA was extracted from gonadal cell populations, gonadal tissues and cultured TCam-2 cells using RNeasy mini kits (Qiagen, Australia) with on-column DNA digestion, as per manufacturer’s instructions. The quality and quantity of total RNAwas assayed using an Agilent 2200 Tape Station (Agilent Technologies, Germany). For each sample, 100 ng total RNA was reverse transcribed using iScript^™^ Advanced cDNA Synthesis Kit (Bio-Rad, USA) according to manufacturer’s instructions. Expression analyses were performed by droplet digital PCR (ddPCR) as described previously [29]; see Table 2) on at least three separate cell or tissue isolations normalized to house-keeping genes (*Mapk1*, *Canx* or *Ppia*) and data were expressed as mean ± standard error of the mean (S.E.M.).

**Table 2:** Digital PCR primer and probe sequences.

Gene	Primer and Probe sets in 5’-3’ orientation	Product size (bp)	Supplier and Catalogue number
***Canx***	Fw:CACATAGGAGGTCTGACAGC Rev:AATTATCTACCCAGGCACCAC P:HEX/TCGGGTCCT/ZEN/CTGGAGCACAAGGCTTT/3IABKFQ	89	IDT/NA
***Mapk1***	Fw:CCTTCAGAGCACTCCAGAAA Rev:AATCTATGCAGTTTGGGATACAAC P:HEX/TCTGCTCTG/ZEN/TACTGTGGATGCCTT/3IABKFQ	93	IDT/NA
***Esrp1***	Fw:TCCTTCTGTCTCTGTACTGACG Rev:GGAATAGAAGCACTCAGGCA P:FAM/AGATCTTGC/ZEN/ATCCCGAGGCTTCC/3IABKFQ	99	IDT/Mm.pt.58.28758847
***Ppia***	Primer sequences not available, FAM RefSeq NM_008907.1, assay location 232, exon boundary 3–4	112	TF/Mm02342429_g1
***Ngn3***	Primer sequences not available, FAM RefSeq NM_009719.6, assay location 493, exon boundary 2–2	73	TF/Mm00437606_s1
***Ccnd1***	Primer sequences not available, FAM RefSeq NM_007631.2, assay location 957, exon boundary 4–5	145	TF/Mm00432360_m1
***Dnah8***	Primer sequences not available, FAM RefSeq NM_013811.3, assay location 2723, exon boundary not available	68	TF/Mm01299527_m1

Sequences of the forward (Fw), reverse (Rev) primers, and internal probe (P) sequences or the catalogue number of proprietary assays, with corresponding product lengths, used for digital RT-PCR analyses. Abbreviations: NA, not applicable; TF = ThermoFisher.

To examine splicing events in cultured TCam-2 cells, RNA was extracted from control and siRNA transfected cells as described above and analyzed by standard RT-PCR and gel electrophoresis to detect splicing of *CTNND1* and *DOCK7*. As *DOCK7* splice variants show alternative splicing of exon 23 (93 nt) we designed primers to exon 22 (5’-GCTAGATCTGCGGTGAGACC-3’) and exon 24 (5’-TCTGTGTGCGAAGACATACG-3’) to detect splice variants and primers to constant exons 2 (5’-TAGTGGTTCTCCCCAACTGC-3’) and 4 (5’-GGATCCATTTCACTTTCTTCAGG-3’) to detect the all *DOCK7* transcripts. Similarly, to detect alternate splicing of *CTNND1* at exons 2 and 3, we designed primers in exon 1 (5’-TGTCTTTCTCAGCACCTTGG-3’) and exon 4 (5’-GTCTTTCAAGGTCAGCATCG-3’) to detect splice variants and primers to the constant exon 5 (5’-CAGATGATGGGACCACTCG-3’) and exon 6 (5’-TCTAGCCCATAAGGCTCTGG-3’) to detect all *CTNND1* transcripts.

### Immunofluorescence

Immunofluorescence, incorporating antigen retrieval with acidic citrate buffer at 100°C, was carried out on paraffin sections of formalin-fixed ovaries and testes from 12-week old mice as described previously [29]. Antibodies used included two rabbit polyclonal anti-human ESRP1 (HPA023719 and HPA023720; Sigma Aldrich) diluted 1:100 and 1:50 for testis and ovary specimens respectively, a goat polyclonal anti-mouse c-KIT (AF1356, R&D system) diluted 1:250, a goat polyclonal anti-human PLZF (AF2944, R &D system) diluted 1:500, a sheep anti-Sox9 antibody (provided by Dr Dagmar Wilhelm) diluted 1:100 and a mouse monoclonal antibody to the nuclear speckle/spliceosome marker, SC35 (ab11826, AbCam) diluted 1:250. Negative controls included sections incubated with non-immune rabbit serum (NIS). Reactivity was visualized using appropriate secondary antibodies conjugated to Alexafluor-488 or Alexafluor-596 (Invitrogen, Australia), diluted 1:500 and imaged using a Zeiss LSM800 confocal microscope (Carl Zeiss, Melbourne, VIC, Australia).

For immunofluorescence of cultured TCam-2 cells, the cells were grown on 13 mm round plastic coverslips (Thermanox^™^) in six-well culture plates as described above. Following siRNA transfection, cells were cultured in RPMI medium for various periods (24, 48, 72 hours) before being fixed in 4% paraformaldehyde in PBS for 20 min, and permeabilized for 20 min in 0.2% TritonX-100 in PBS. After blocking with 10% FCS in PBS for 30 min, cells were incubated overnight at 4°C with anti-ESRP1 primary antibody (HPA023719; Sigma) diluted 1:50 in blocking buffer. Antibody binding was detected by incubating with 1:500 AlexaFluor 488-conjugated donkey anti-rabbit IgG (Invitrogen A-21052) for 1h. Cells were counter-stained with 1 μg/ml Hoechst dye and 1 μg/ml Phalloidin-TRITC (Sigma) for 1h. Matching plastic coverslips for each treatment were mounted onto 50 mm square glass coverslips and visualized using confocal microscopy as described above.

### Statistical analyses

For analyses of the ddPCR data, application of a Shapiro-Wilks normality test indicated the data fitted a Gaussian distribution. Therefore, where at least three biological replicates were available, a one-way analysis of variance with a Tukey’s post-hoc analysis and α = 0.05 was employed to determine significant differences among groups. All statistical analyses and generation of graphs were performed using GraphPad Prism (La Jolla CA, USA).

## Results

### *Esrp1* is expressed in fetal germ but not somatic cells

**Fig 1:**
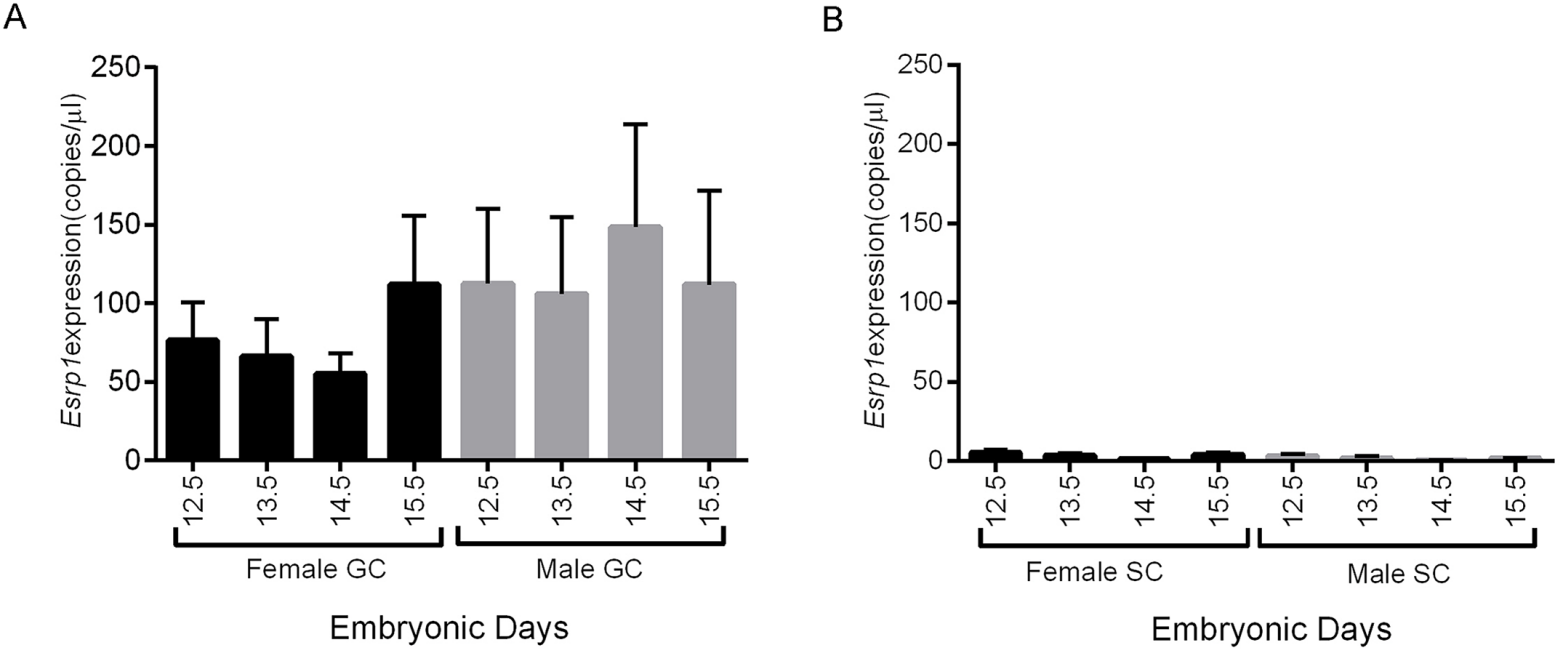
*Esrp1 is* expressed in developing male and female germ cells but not somatic cells. Histograms show *Esrp1* RNA expression, measured by RT-ddPCR, in FACS sorted (A) germ (GC) and (B) somatic (SC) cells, isolated from E12.5-E15.5 mouse embryos expressing an *Oct4* (*Pou5f1*)-GFP transgene. Gene expression is expressed as copies/μl and normalised against *Mapk1* expression in germ cells and against *Canx* expression in somatic cells. Data are presented as mean ± S.E.M of three independent samples at each age. Transcripts for *Esrp1* were detected in male and female germ cells but not in sorted somatic cells. No significant differences in expression were detected in *Esrp1* expression with age in male or female germ cells (n = 3 for each group; One-way ANOVA, p>0.05). Abbreviations: GC, germ cells; SC, somatic cells.

To examine *Esrp1* expression specifically in fetal germ and somatic cells of murine gonads, we took advantage of the *Pou5f1-GFP* (*Oct4-GFP*) transgenic mouse line to isolate male and female gonadal (GFP^+^) and somatic (GFP^-^) cells at various stages of development by FACS [28]. Using ddPCR we found that *Esrp1* is highly expressed in female and male germ cells but not somatic cells from E12.5 to E15.5 (Fig 1). These data are consistent with published microarray data showing detectable expression of *Esrp1* in only male and female germ cells from E11.5 –E14.5 [35]. To examine if ESRP1 protein could be detected in fetal gonads, immunofluorescence experiments were conducted on mouse E12.5-E14.5 male and female gonadal sections. However, little or no specific reactivity could be detected in somatic or germ cells and if ESRP1 protein is produced in fetal germ cells it is below the level of detection via immunofluorescence.

### *Esrp1* is upregulated in adult spermatogonia

To examine the expression of *Esrp1* during postnatal male gametogenesis, we utilized a BSA gradient to separately enrich gonocytes/spermatogonia (at P1), spermatogonia (at P8-10), pachytene spermatocytes and round spermatids (at 8 weeks) as described previously [30–32].

**Fig 2:**
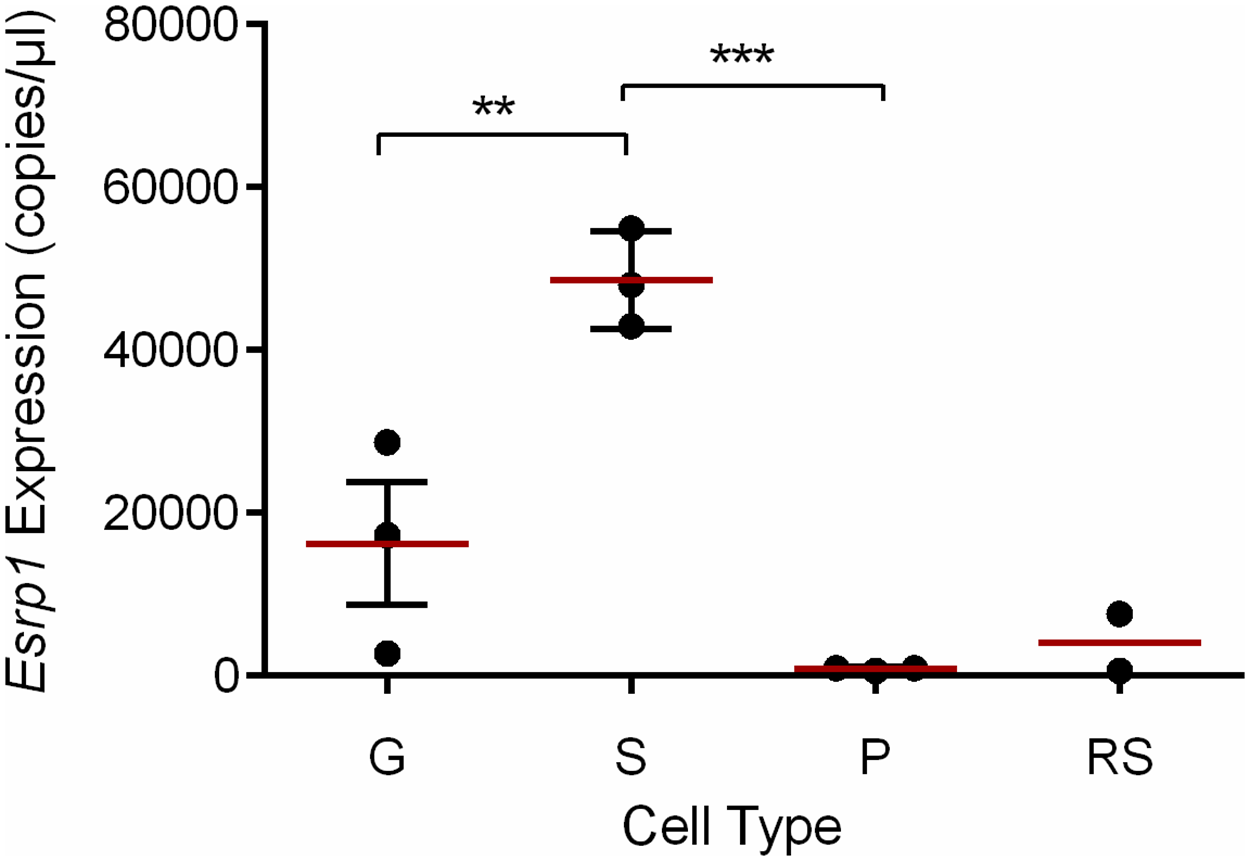
*Esrp1* is highly expressed in spermatogonia in the adult testis. RT-ddPCR of isolated germ cells from postnatal mouse testes show that *Esrp1* is highly expressed in spermatogonia (S), weakly expressed in gonocytes/spermatogonia (G) and barely detectable in pachytene spermatocytes (PS) and round spermatids (RS). Expression data are expressed as copies/μl, normalised against *Cyclophilin A* expression, and as mean ± S.E.M of three independent samples (except for Round Spermatids n = 2). Individual data are shown as black circles, mean as a red line and the error bars in black. Asterisks indicate significance differences by ANOVA and Tukey’s post-hoc analysis (**, p<0.01; ***, p<0.001).

Previous studies have demonstrated that this method can be used to isolate gonocyte- and spermatogoinal-enriched popluations, expressing specific marker (*Pou5f1*, *Nanog*, *Ngn3*, *Plzf)* profiles [32]. Using RT-ddPCR we confirmed (S1 Fig) that each population was specifically enriched for gonocytes (*Ccnd1*), spermatogonia (*Ngn3)* and pachytene spermatocytes (*Dnah8)*. In addition, we have recently shown, using the same RNA samples as used in this study, that the round spermatid sample uniquely expressed *Tob1* [29]. The RT-ddPCR analysis indicated that *Esrp1* was most strongly expressed in cell populations enriched for spermatogonia. Greatly reduced expression was detected in pachytene spermatocytes and round spermatids (Fig 2).

**Fig 3:**
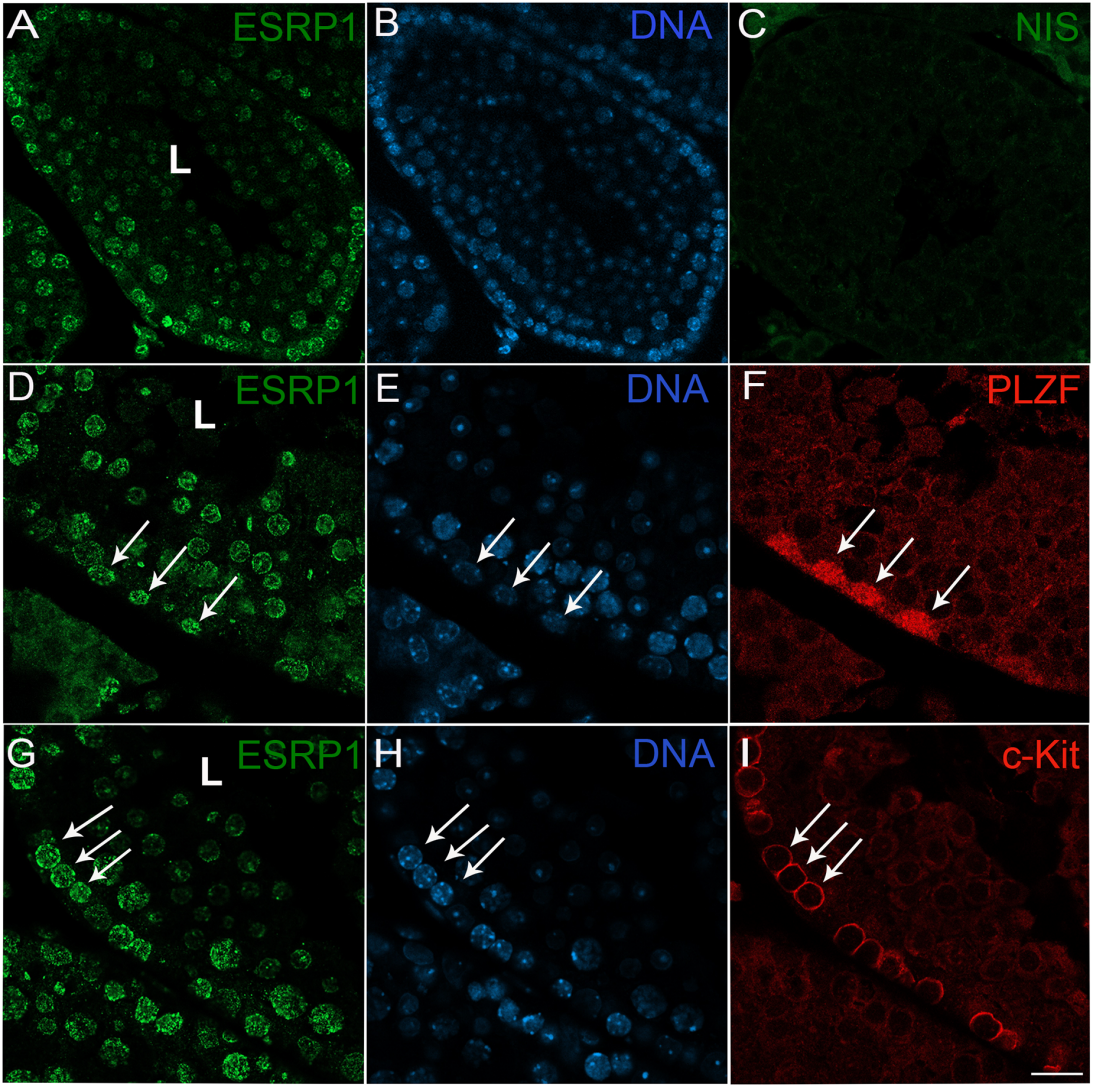
ESRP1 is expressed in type A and type B spermatogonia. Double labelling immunofluorescence showed that ESRP1 (antibody HPA023719) is detected in both Type A (PLZF^+^) and Type B (c-Kit^+^) spermatogonia, Representative sections of seminiferous tubules were labelled with antibodies to ESRP1 (A, D, G), PLZF (F), c-Kit (I) and Hoechst nuclear stain (B, E, H). ESRP1 staining in spermatogonia is present as speckled nuclear reactivity (A, D, G). Arrows indicate double-labelled cells in each image series (D-F; G-I). Very little non-specific labelling was detected when sections were incubated with non-immune serum (NIS; C). Scale bars: 34.5 μm (A-B); 20.5 μm (C); 10.5 μm (D-F); 18 μm (G-I).

**Fig 4:**
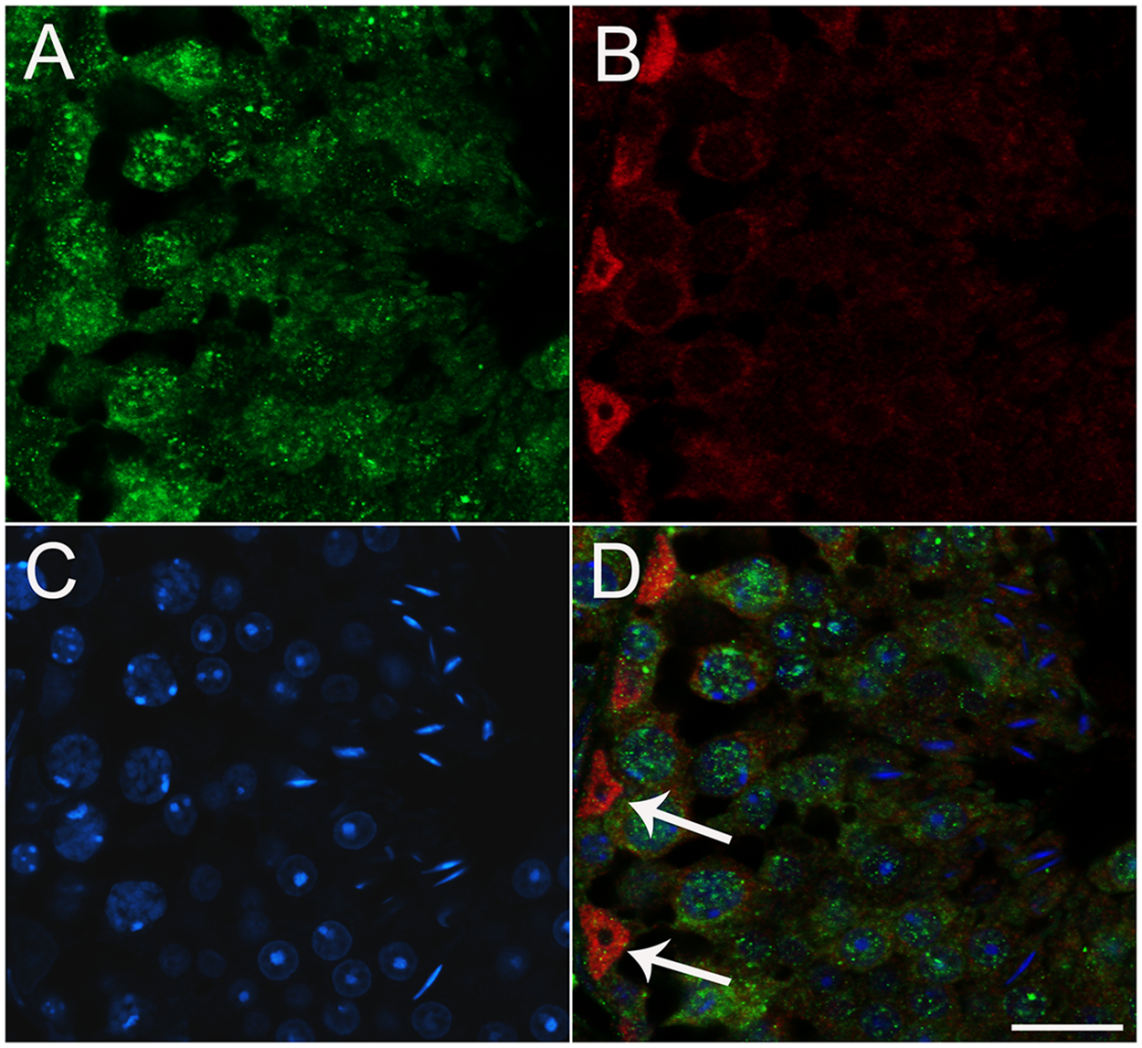
ESRP1 is not localized to somatic (Sertoli) cells. Double labelling immunofluorescence experiments with an antibody to Sox9 (A) showed that ESRP1 (B; antibody HPA023719) exhibits minimal labelling of somatic Sertoli cells in the testis. Scale bar: 20 μm all images.

To confirm ESRP1 expression in adult spermatogonia, we used immunofluorescence with a specific antibody for ESRP1 (Fig 3). Consistent with the ddPCR data, strong nuclear immunostaining for ESRP1 was detected in spermatogonia that reside close to the basal lamina of the seminiferous tubules (arrows, Fig 3A, 3D and 3G), with expression also observed in differentiating spermatocytes and spermatids. The presence of immunostaining in these cells suggests that the greatly lower levels of mRNA (mean ± SEM; 852 ± 125 copies/μl; see Fig 2) are still sufficient to allow for translation of the protein. This level of expression is more than an order of magnitude higher than that of Ngn3 expression in spermatogonia (S1 Fig), indicating that these lower levels of mRNA expression can still result in readily detectable protein levels. The lack of staining with non-immune serum (Fig 3C) combined with western blot and immunofluorescence analyses of *ESRP1*-siRNA transfected TCam-2 cells (S2 Fig) indicate that the antibody (HPA023719) is specific. Moreover, similar patterns of reactivity were detected with another antibody to ESRP1 (S3 Fig). To determine if ESRP1 expression was restricted to a specific type of spermatogonial cell, we performed a co-labelling experiment with PLZF and c-KIT antibodies, which mark type A_s_-A_al_ and type A_1_-B spermatogonia respectively [36, 37]. The results showed that ESRP1 is expressed in both Type A and Type B (Fig 3D–3I) spermatogonia. To confirm that ESRP1 was not associated with somatic cells, we performed co-localization studies with Sox9, a marker for testicular somatic Sertoli cells. Consistent with the ddPCR data indicating little or no expression of *Esrp1* in somatic cells (Fig 1), ESRP1 showed little to no co-localization with Sox9 in the somatic Sertoli cells (Fig 4).

**Fig 5:**
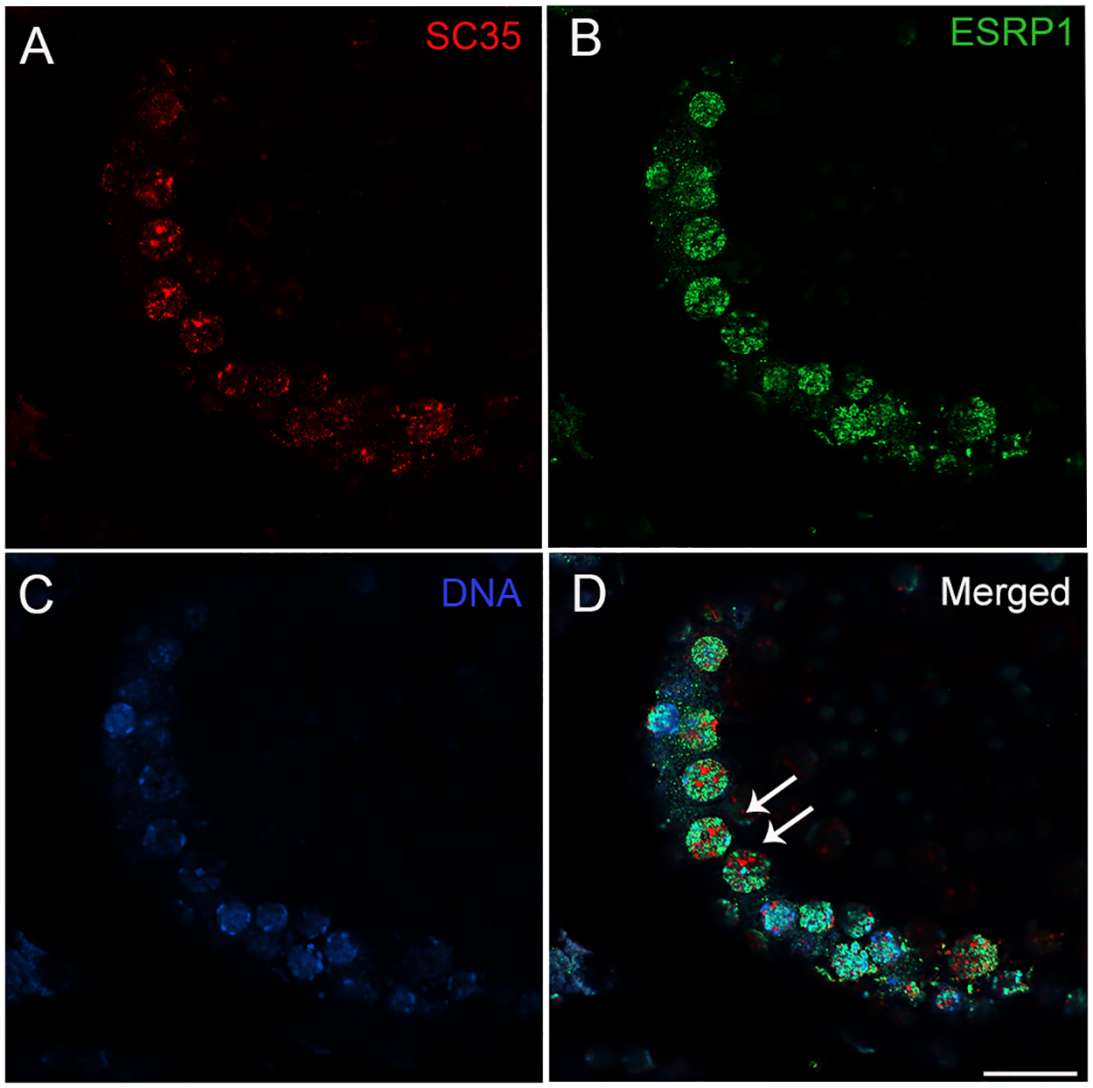
Nuclear ESRP1does not co-localize with the spliceosome marker, SC35. Double labelling immunofluorescence experiments of ESRP1 (antibody HPA023719) with an antibody to SC35, a marker for the spliceosome (A), showed that the nuclear ESRP1+ speckles (B) do not overlap with SC35+ nuclear speckles (D). Scale bar: 20 μm all images.

The nuclear speckled pattern of reactivity seen in the spermatogonia suggested that ESRP1 may be localized to the spliceosome. To investigate this further, we examined whether ESRP1 co-localized with SC35, a well-known constituent of nuclear speckles and pre-mRNA splicing [38, 39]. Surprisingly, ESRP1 did not co-localize to SC35^+^ nuclear speckles (Fig 5).

### ESRP1 regulates splicing in a fetal germ cell line (TCam-2 cells)

**Fig 6:**
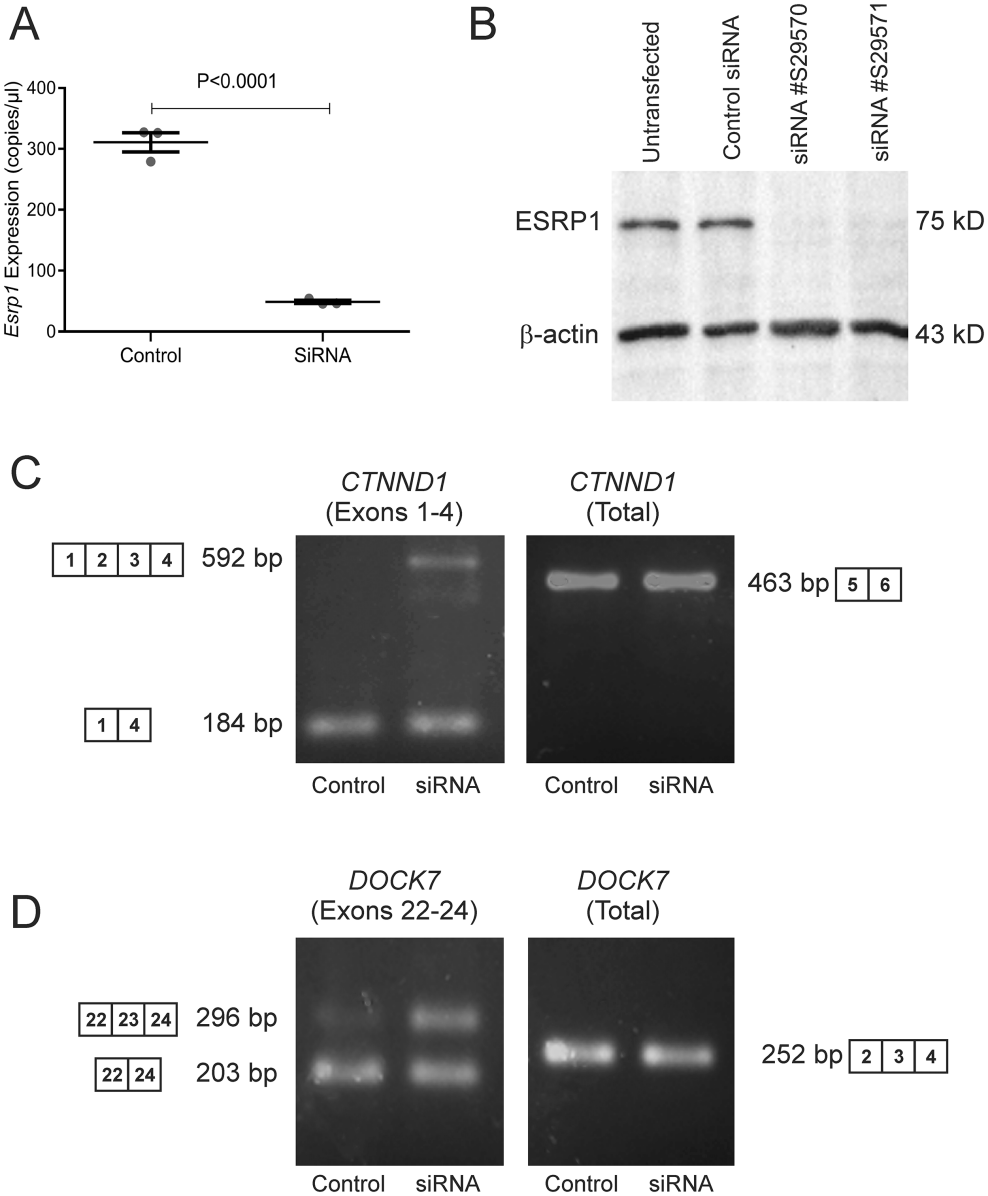
ESRP1-mediated alternative splicing in TCam-2 cells. **A.** TCam-2 cells transfected with siRNA constructs for *ESRP1* displayed a dramatic (~85%) and significant (n = 3 each group; p<0.0001, Student’s *t*-test) decrease in expression of *ESRP1* mRNA. Similar data were obtained in two independent experiments. **B.** By western blot analysis, a distinct 75kD band for ESRP1 was detected in non-transfected and control siRNA-transfected TCam2 cells but was not detectable in cells transfected with either siRNA construct. Similar data were obtained in two independent experiments. **C.** RT-PCR using primers specific to splice variants of *CTNND1* and *DOCK7* showed that loss of ESRP1 expression promoted inclusion of exons 2–3 in *CTNND1* transcripts and inclusion of exon 23 in *DOCK7* transcripts. By contrast no changes were detected for the constant exons in these genes. The splicing pattern and band size are illustrated for each transcript variant.

To investigate if ESRP1 can modulate splicing of target genes in fetal germ cells we examined the effects of siRNA knockdown of *ESRP1* in vitro, using the seminoma-derived TCam-2 cell line, which has been shown to have characteristics of fetal germ cells (Young et al., 2011). To first determine the lability of the ESRP1 protein in Tcam2 cells we conducted a cycloheximde chase analysis and found that ESRP1 protein remains stable in these cells for at least 24 hours. By 48 hours, small amounts of the protein are still detected by western blot, but by 72 hours no protein was detected (S4 Fig). We therefore conducted our analyses of siRNA knockdown cells 72 hours post-transfection. Transfection of these cells with two independent siRNA constructs resulted in efficient knock-down of *ESRP1* mRNA and protein, as determined by RT-ddPCR (Fig 6A), and by western blot (Fig 6B). As previous studies [40] demonstrated that exons 2 and 3 of *CTNND1* and exon 23 of *DOCK7* are direct targets of ESRP1-mediated splicing, we designed primers to detect the alternatively spliced and constant exons of these transcripts and examined their expression by RT-ddPCR in cells treated with or without siRNA. Consistent with previous reports that ESRP1 mediates exclusion of exons2 and 3 in *CTNND1* transcripts, we detected a shift from the variants lacking exons 2–3 to variants that include these exons in cells treated with *ESRP1* siRNA (Fig 6C). Similarly, greater inclusion of exon 23 in *DOCK7* transcripts was detected, whereas the expression of the constant exons for both genes remained unchanged (Fig 6C).

## Discussion

In this study we documented the expression of *Esrp1* during gonadal development and in differentiating cells of the adult gonads. We observed that in mouse fetal gonads, *Esrp1* mRNA was specifically detected in germ cells of either sex and absent from somatic gonadal cells. *Esrp1* mRNA was also present in a gonocyte-enriched cell fraction and in all germ cells examined in the postnatal testis; however, it was found at much higher levels in spermatogonia than in pachytene spermatocytes or round spermatids. Consistent with mRNA levels, ESRP1 protein expression was detected in cells at the periphery of seminiferous tubules, co-staining with PLZF and c-Kit, indicating that ESRP1 is present in type A and B spermatogonia but not in Sox9^+^ Sertoli cells. Protein expression was also detected in spermatocytes and round spermatids, suggesting that the level of mRNA expression in these cells can sustain detectable protein production. This raises the possibility that ESRP1 plays a continuing role in splicing of pre-meiotic transcripts in these more differentiated cells. Spermatogenesis is characterized by a variety of post-transcriptional regulatory events and alternative splicing may plausibly regulate these later events [41].

While it is unclear precisely where splicing occurs within the nucleus, it is becoming clear that constitutive splicing occurs most commonly as co-transcriptional splicing of the pre-mRNA and that alternate splicing occurs more commonly as a post-transcriptional process [42]. Spliceosome assembly involves the sequential recruitment of U1, U2, U4, U5 and U6 snRNAs and various small ribonucleoproteins into a macromolecular complex at the pre-mRNA splice sites, [42]. Studies with the U2-associated protein, SF3b155, have shown that 80% of pre-mRNA splicing, marked by anti-phospho- SF3b155 antibodies, occurs co-transcriptionally, whereas only 10–20% of splicing occurs post-transcriptionally in nuclear speckles, which are positive for SC35 [43]. Consistent with previous findings indicating that ESRP1 is a splicing factor, ESRP1 was localized to numerous small nuclear speckles in spermatogonia. However, the lack of ESRP1 co-localization with SC35 suggests that ESRP1 is more likely to be involved in constitutive, co-transcriptional rather than post-transcriptional, alternate splicing events in SC35^+^ speckles during spermatogonial self-renewal and differentiation. Despite the lack of co-localization with SC35, siRNA experiments indicated that down-regulation of *ESRP1* expression resulted in expected changes in the splicing of two known ESRP1 target pre-mRNAs (*CTNND1* and *DOCK7*). These data suggest that ESRP1 plays a role in regulating alternative splicing in male germ cells *in vitro*. Whether this extends to regulating these ESRP1 target genes in the testis *in vivo* remains to be determined. The standard spliced form of *CD44* mRNA is expressed in the testis but not the splice variant, *CD44v6*, mediated by ESRP1 [44]. Similarly, while FGFR2 is expressed in spermatogonia, spermatocytes and spermatids [45], it is not known if this is the IIIC or the IIIB isoform.

Transcriptomic analysis of mouse spermatogenesis has identified over 13,000 alternative splicing events [8], indicating that alternative splicing is a key driver of cell differentiation events during spermatogenesis. However, while ESRP1 is known as a an important regulator of alternative splicing in epithelial tissues, in the testis it appears not to function in nuclear speckles associated with post-transciptional alternate splicing. Our data cannot exclude an alternate splicing role for ESRP1 in the testis and combined expression and bioinformatic analyses may yet yield information about potential ESRP1 target genes in the testis. Additionally, analyses of spermatogenesis and spermiogenesis in *Esrp1*^*-/-*^ mice will be required to determine the precise role of *Esrp1* in spermatogonia.

## Supporting information

S1 Fig Analysis of separated germ cell populations. Droplet digital RT-PCR analyses of BSA gradient-separated cells from mouse testes confirmed that the isolated populations of cells are enriched for spermatogonia (S; *Ngn3* and *Ccnd1* expressing) and pachytene spermatids (P; *Dnah8* expressing). Gonocytes (G), like spermatogonia (S), express *Plzf* but express very low levels of *Ccnd1*. In another study we have shown that round spermatids express elevated *Tob1* [29]. Statistical analyses: One-way ANOVA with Tukey’s post-hoc analyses; n = 3 in all cases, except RS (n = 2); *, p<0.05; ****, p<0.0001.
(TIF)

S2 Fig siRNA knockdown of Esrp1 demonstrates antibody specificity. Immunofluorescence of TCam-2 cells transfected with negative control-siRNA (**A-D**) or with *ESRP1*-siRNA (**E-H**). In control cells, ESRP1 (**A**) showed a granular nuclear immunofluorescent staining (HPA023719; Sigma; 1:100), which was greatly depleted in siRNA-treated cells (E). Cells were counterstained with phalloidin to stain filamentous cortical actin (**B, F**) and Hoechst dye to label cell nuclei (**C, G**). Merged images are shown in **D** and **F**. Scale bar, 20 μm for all images.
(TIF)

S3 Fig Immunostaining with a second ESRP1 antibody shows a similar expression pattern. ESRP1 immunofluorescence in adult mouse testis using another antibody (Sigma-Aldrich, HPA023720; Lot: 3070388) showed similar nuclear staining in spermatogonia (**A, C, arrows**) to that observed with HPA023719 (Fig 3). Non-immune IgG (**D, F**) showed no reactivity in the seminiferous tubules but did show but nonspecific labelling in the interstitial Leydig cells (*). Section were counter-stained with Hoechst dye to label nuclei (**B, E**) and merged images are shown in (**C, F**). Scale bar: A-C, 30 μm; D-F, 20 μm.
(TIF)

S4 Fig ESRP1 protein is depleted 72 hours after blocking translation. Immunoblot of cycloheximide (CHX) chase experiment in TCam-2 cells showing stability of ESRP1 protein after arrest of protein synthesis. ESRP1 protein (75kD) was still detected weakly after 48 hours but was absent by 72 hours. Beta-actin (43 kD) was used as a loading control and was present in all samples. ESRP1 antibody HPA023719)
(TIF)

S1 File Supplementary legends. (DOCX)
