# Supplementary figures and images for: Esrp1 is a marker of mouse fetal germ cells and differentially expressed during spermatogenesis
Source: PLoS One. 2018 Jan 11;13(1):e0190925. doi: 10.1371/journal.pone.0190925 (PMC5764326; doi:10.1371/journal.pone.0190925)

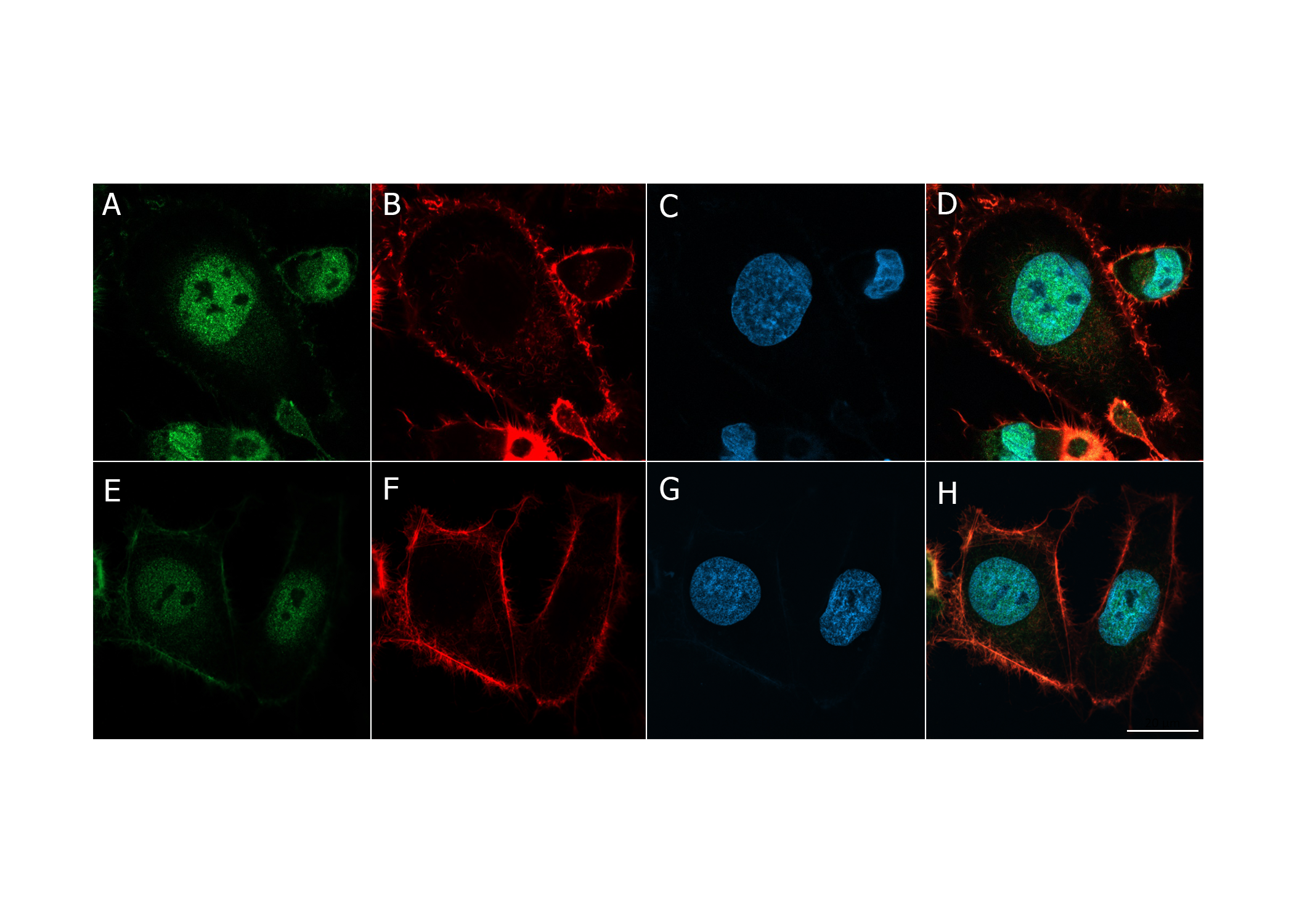

Supplement: S2 Fig — Immunofluorescence of TCam-2 cells transfected with negative control-siRNA (A-D) or with ESRP1-siRNA (E-H). In control cells, ESRP1 (A) showed a granular nuclear immunofluorescent staining (HPA023719; Sigma; 1:100), which was greatly depleted in siRNA-treated cells (E). Cells were counterstained with phalloidin to stain filamentous cortical actin (B, F) and Hoechst dye to label cell nuclei (C, G). Merged images are shown in D and F. Scale bar, 20 μm for all images. (TIF) [file pone.0190925.s002.tif]

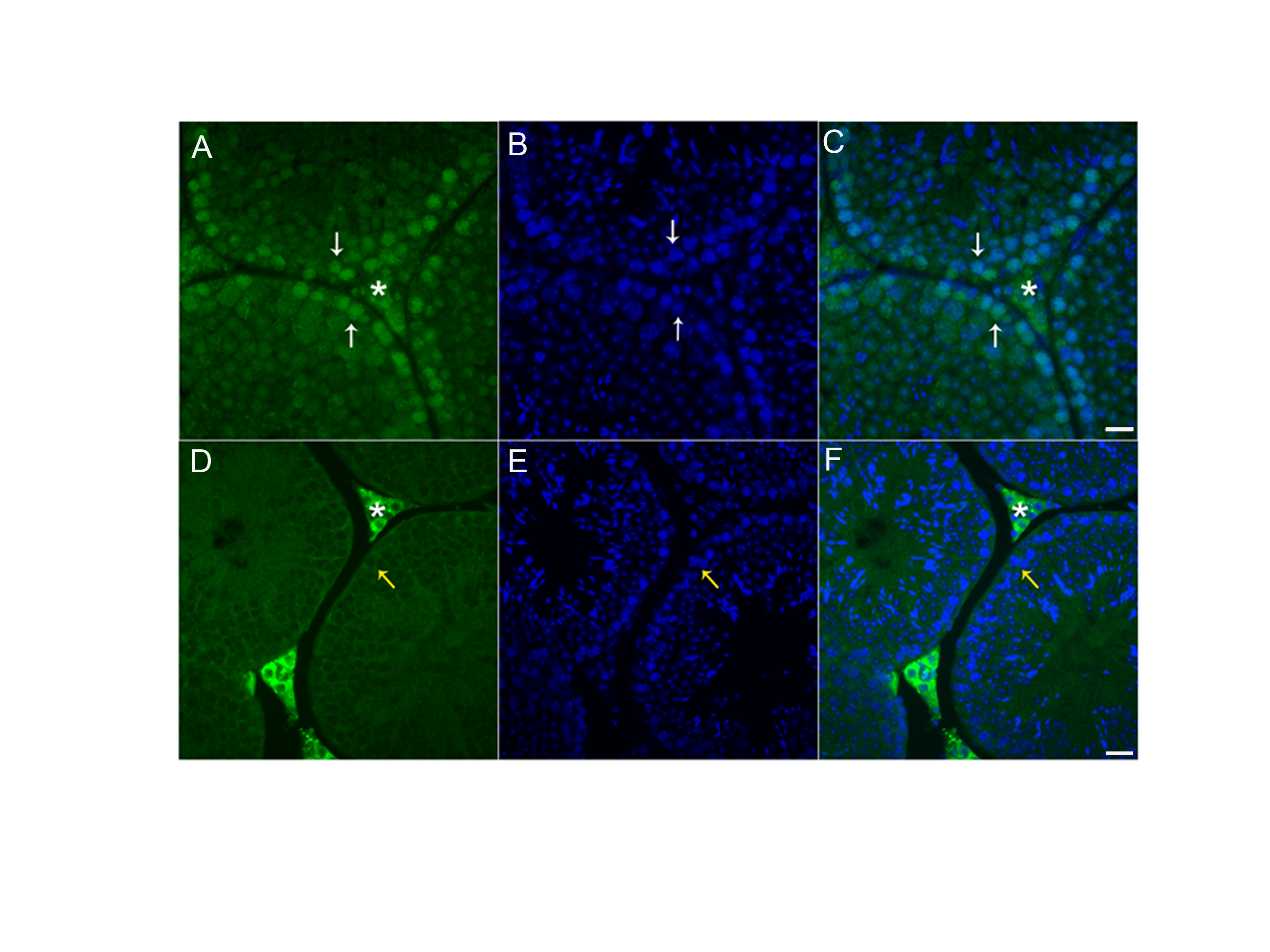

Supplement: S3 Fig — ESRP1 immunofluorescence in adult mouse testis using another antibody (Sigma-Aldrich, HPA023720; Lot: 3070388) showed similar nuclear staining in spermatogonia (A, C, arrows) to that observed with HPA023719 (Fig 3). Non-immune IgG (D, F) showed no reactivity in the seminiferous tubules but did show but nonspecific labelling in the interstitial Leydig cells (*). Section were counter-stained with Hoechst dye to label nuclei (B, E) and merged images are shown in (C, F). Scale bar: A-C, 30 μm; D-F, 20 μm. (TIF) [file pone.0190925.s003.tif]

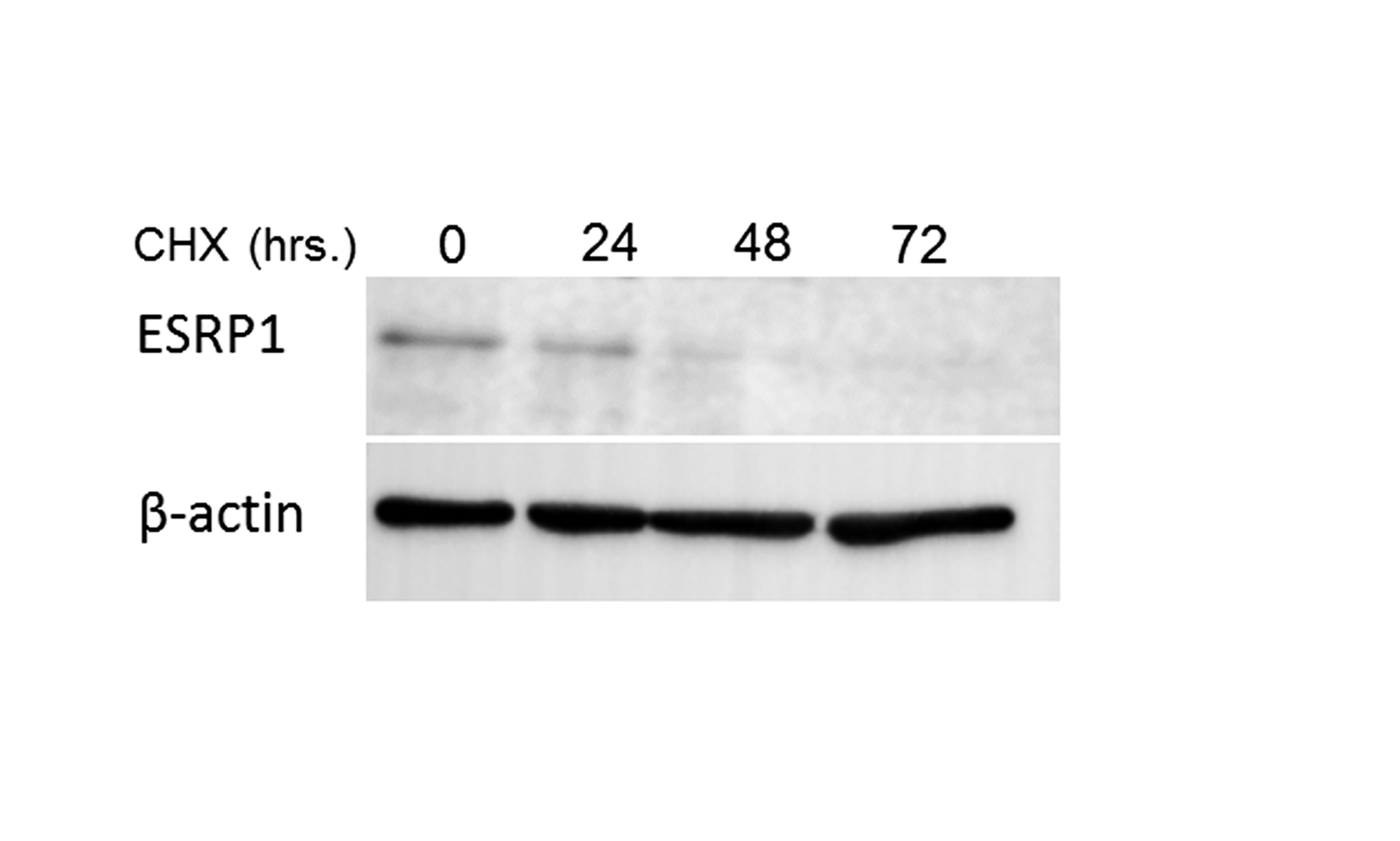

Supplement: S4 Fig — Immunoblot of cycloheximide (CHX) chase experiment in TCam-2 cells showing stability of ESRP1 protein after arrest of protein synthesis. ESRP1 protein (75kD) was still detected weakly after 48 hours but was absent by 72 hours. Beta-actin (43 kD) was used as a loading control and was present in all samples. ESRP1 antibody HPA023719) (TIF) [file pone.0190925.s004.tif]
