# Supplementary material for: Esrp1 is a marker of mouse fetal germ cells and differentially expressed during spermatogenesis
Source: PLoS One. 2018 Jan 11;13(1):e0190925. doi: 10.1371/journal.pone.0190925 (PMC5764326; doi:10.1371/journal.pone.0190925)
Supplement: S1 File — (DOCX) [file pone.0190925.s005.docx]

**Figure S1**

Droplet digital RT-PCR analyses of BSA gradient-separated cells from mouse testes confirming that the isolated populations of cells are enriched for spermatogonia (S; *Ngn3* and *Ccnd1* expressing) and pachytene spermatids (P; *Dnah8* expressing)*.*  Gonocytes (G), like spermatogonia (S), express *Plzf* but express very low levels of *Ccnd1.* In another study we have shown that round spermatids express elevated *Tob1* [1]. Statistical analyses: One-way ANOVA with Tukey’s post-hoc analyses; n=3 in all cases, except RS (n=2); *, p<0.05; ****, p<0.0001.

**Figure S2**

Immunofluorescence of TCam-2 cells transfected with negative control-siRNA (**A-D**) or with *ESRP1*-siRNA (**E-H**). In control cells, ESRP1 (**A**) showed a granular nuclear immunofluorescent staining (HPA023719; Sigma; 1:100), which was greatly depleted in siRNA-treated cells (E). Cells were counterstained with phalloidin to stain filamentous cortical actin (**B, F**) and Hoechst dye to label cell nuclei (**C, G**). Merged images are shown in **D** and **F**. Scale bar, 20 μm for all images.

**Figure S3**

ESRP1 immunofluorescence in adult mouse testis using another antibody (Sigma-Aldrich, HPA023720; Lot: 3070388) showed similar nuclear staining in spermatogonia (**A, C, arrows**) to that observed with HPA023719 (Fig. 3). Non-immune IgG (**D, F**) showed no reactivity in the seminiferous tubules but did show but nonspecific labelling in the interstitial Leydig cells (*). Section were counter-stained with Hoechst dye to label nuclei (**B, E**) and merged images are shown in (**C, F**). Scale bar: A-C, 30 μm; D-F, 20 μm.

**Figure S4**

Immunoblot of cycloheximide (CHX) chase experiment in TCam-2 cells showing stability of ESRP1 protein after arrest of protein synthesis. ESRP1 protein (75kD) was still detected weakly after 48 hours but was absent by 72 hours. Beta-actin (43 kD) was used as a loading control and was present in all samples. ESRP1 antibody HPA023719)

1. Shapouri F, Saeidi S, de Iongh RU, Casagranda F, Western PS, McLaughlin EA, et al. Tob1 is expressed in developing and adult gonads and is associated with the P-body marker, Dcp2. Cell Tissue Res. 2016;364(2):443-51. doi: 10.1007/s00441-015-2328-z. PubMed PMID: 26662055.
